# 2-Amino-6-methyl­pyridinium *trans*-di­aqua­dioxalatochromate(III) monohydrate

**DOI:** 10.1107/S1600536813022058

**Published:** 2013-08-10

**Authors:** Rihab Dridi, Saoussen Namouchi Cherni, Mohamed Faouzi Zid, Ahmed Driss

**Affiliations:** a Laboratoire de Matériaux et Cristallochimie, Département de Chimie, Faculté des Sciences, 2092 El Manar, Tunis, Tunisia

## Abstract

In the title compound, (C_6_H_9_N_2_)[Cr(C_2_O_4_)_2_(H_2_O)_2_]·H_2_O, the Cr^III^ atom adopts a slightly distorted octa­hedral coordination environment defined by two chelating oxalate ligands in the equatorial plane and two water mol­ecules in axial positions. A three-dimensional network is generated by inter­molecular N—H⋯O and O—H⋯O hydrogen-bonding interactions involving the cation, the complex anion and the lattice water molecule.

## Related literature

For general background to the coordination chemistry of oxalates, see: Martin *et al.* (2007 ▶). For the structural characterization of organic–inorganic salts containing the [Cr(C_2_O_4_)_2_(H_2_O)_2_]^−^ anion, see: Bélombé *et al.* (2009 ▶); Nenwa *et al.* (2010 ▶); Chérif *et al.* (2011 ▶); Chérif, Abdelhak *et al.* (2012 ▶); Chérif, Zid *et al.* (2012 ▶). For C—O bond lengths in oxalate anions, see: Marinescu *et al.* (2000 ▶). For geometric parameters of the 2-amino-6-methyl­pyridinium cation, see: Fun *et al.* (2008 ▶, 2009 ▶, 2010 ▶); Jebas *et al.* (2009 ▶); Quah *et al.* (2008 ▶); Ramesh *et al.* (2010 ▶); Rotondo *et al.* (2009 ▶); Pan *et al.* (2008 ▶). For discussion of hydrogen bonding, see: Blessing (1986 ▶); Brown (1976 ▶).
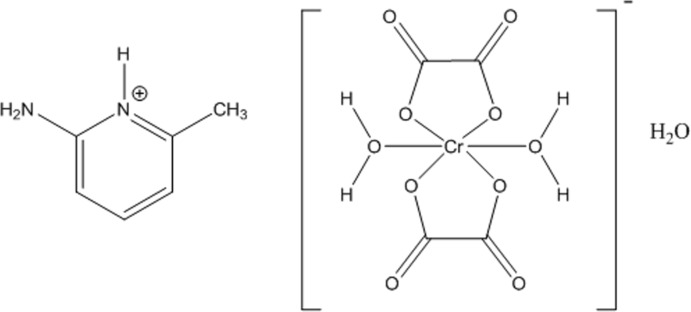


## Experimental

### 

#### Crystal data

(C_6_H_9_N_2_)[Cr(C_2_O_4_)_2_(H_2_O)_2_]·H_2_O
*M* *_r_* = 391.24
Monoclinic, 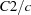

*a* = 18.572 (3) Å
*b* = 11.025 (2) Å
*c* = 14.975 (3) Å
β = 96.28 (2)°
*V* = 3047.8 (10) Å^3^
*Z* = 8
Mo *K*α radiation
μ = 0.81 mm^−1^
*T* = 298 K
0.56 × 0.42 × 0.33 mm

#### Data collection

Enraf–Nonius CAD-4 diffractometer
Absorption correction: ψ scan (North *et al.*, 1968 ▶) *T*_min_ = 0.679, *T*_max_ = 0.764
4604 measured reflections
3312 independent reflections
2896 reflections with *I* > 2σ(*I*)
*R*_int_ = 0.037
2 standard reflections every 120 min intensity decay: 3.2%

#### Refinement

*R*[*F* ^2^ > 2σ(*F* ^2^)] = 0.040
*wR*(*F* ^2^) = 0.112
*S* = 1.07
3312 reflections
218 parameters
H-atom parameters constrained
Δρ_max_ = 0.65 e Å^−3^
Δρ_min_ = −0.45 e Å^−3^

### 

Data collection: *CAD-4 EXPRESS* (Duisenberg, 1992 ▶; Macíček & Yordanov, 1992 ▶); cell refinement: *CAD-4 EXPRESS*; data reduction: *XCAD4* (Harms & Wocadlo, 1995 ▶); program(s) used to solve structure: *SHELXS97* (Sheldrick, 2008 ▶); program(s) used to refine structure: *SHELXL97* (Sheldrick, 2008 ▶); molecular graphics: *DIAMOND* (Brandenburg & Putz, 1999 ▶); software used to prepare material for publication: *WinGX* (Farrugia, 2012 ▶).

## Supplementary Material

Crystal structure: contains datablock(s) I, New_Global_Publ_Block. DOI: 10.1107/S1600536813022058/mw2113sup1.cif

Structure factors: contains datablock(s) I. DOI: 10.1107/S1600536813022058/mw2113Isup2.hkl

Additional supplementary materials:  crystallographic information; 3D view; checkCIF report

## Figures and Tables

**Table 1:** Hydrogen-bond geometry (Å, °)

*D*—H⋯*A*	*D*—H	H⋯*A*	*D*⋯*A*	*D*—H⋯*A*
O1—H3⋯O7^i^	0.83	1.82	2.643 (2)	171
O1—H4⋯O10^ii^	0.83	1.87	2.681 (2)	165
O2—H2⋯O11^iii^	0.83	1.78	2.611 (2)	173
O2—H10⋯O8^iv^	0.83	1.85	2.675 (2)	172
N1—H7⋯O9^v^	0.93	1.90	2.829 (2)	174
N2—H5⋯O7^vi^	0.93	2.16	2.928 (3)	139
N2—H13⋯O10^v^	0.93	2.08	2.976 (3)	162
O11—H1⋯O8^i^	0.83	2.17	2.983 (2)	167
O11—H11⋯O6	0.83	2.03	2.845 (2)	169

Symmetry codes: (i) 
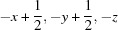
; (ii) 
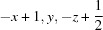
; (iii) 
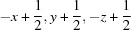
; (iv) 
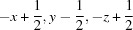
; (v) 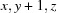; (vi) 
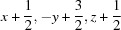
.

**Fig. 1.**
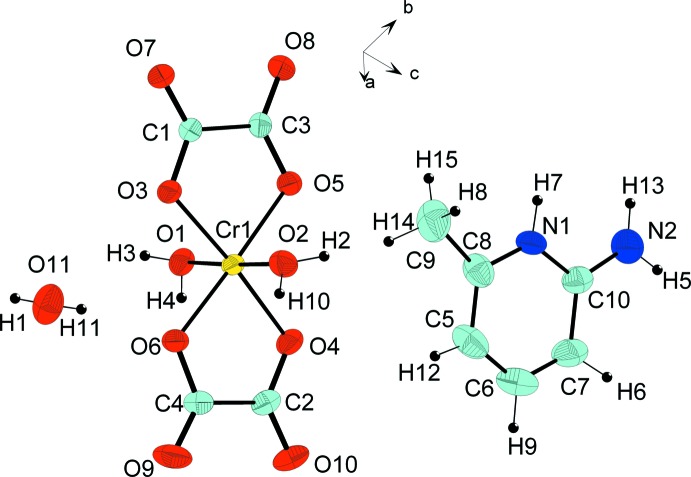
The asymmetric unit of (C6H9N2)[Cr(H2O)2(C2O4)2].H2O.

**Fig. 2.**
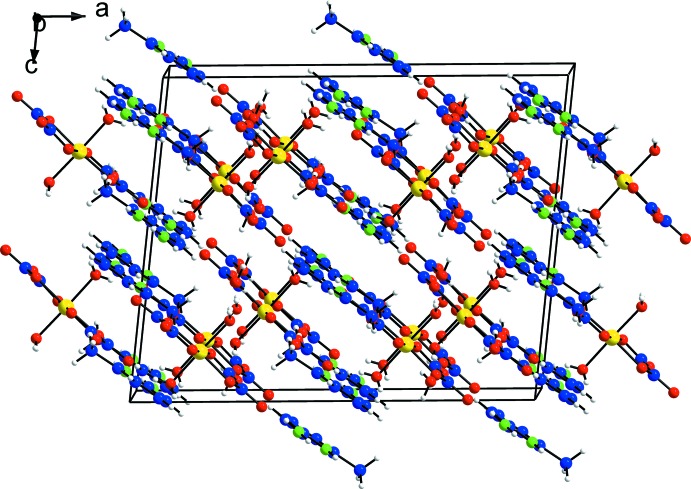
Projection of the (C6H9N2)[Cr(H2O)2(C2O4)2].H2O structure along the b axis.

**Fig. 3.**
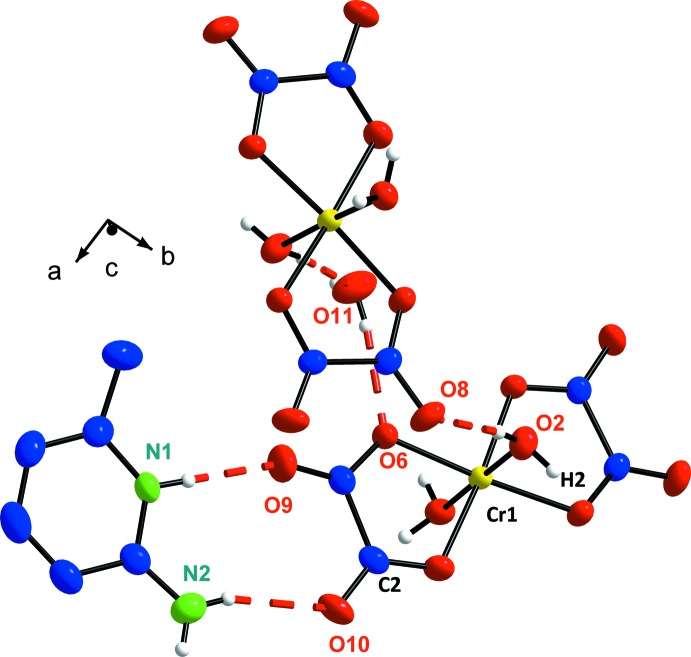
Closeup view of the hydrogen bonding (dotted lines). H atoms not involved in hydrogen bonding have been omitted for clarity.

## supplementary crystallographic information

### 1. Comment

The coordination chemistry of oxalates (C_2_O_4_^2-^) continues to receive considerable attention, largely due to the ability of this ion to act as a remarkably flexible ligand system in complexations with a wide range of metal ions (Martin *et al.*, 2007). Recently, certain hybrid organic-inorganic salts were reported of formula A[Cr(H_2_O)_2_(C_2_O_4_)_2_].xH_2_O (A^+^ = aromatic iminium cation, 0 ≤*x*≤ 1) (Bélombé *et al.*, 2009; Nenwa *et al.*, 2010; Chérif *et al.*, 2011; Chérif, Abdelhak *et al.*, 2012; Chérif, Zid *et al.*, 2012). These salts form different crystalline structures depending on the nature of the substituent and/or substitution position on the ring of the pyridinium cation (Chérif, Abdelhak *et al.*, 2011, 2012; Chérif, Zid *et al.*, 2012; Nenwa *et al.*, 2010). In a continuation of these studies, we report the structure of (amp)[Cr(C_2_O_4_)_2_(H_2_O)_2_].H_2_O (amp = 2-amino-6-methylpyridinium ion) with the anion having *trans*-geometry. The asymmetric unit of the title compound consists a [Cr(C_2_O_4_)_2_(H_2_O)_2_]^-^ anion, a (C_6_H_9_N_2_)^+^ cation and one uncoordinated water molecule (Figure 1). The coordination environment of the chromium(III) is distorted octahedral since the O—Cr—O angles vary from 82.48 (6)° to 101.02 (6)° while the four Cr—O (ox) distances are slightly shorter than the two Cr—O(water) distances. Similar patterns of distortion have been observed in homologous salts involving quinolinium (C_9_H_8_N)^+^, 4-dimethylaminopyridinium (C_7_H_11_N_2_)^+^ and 4-aminopyridinium (C_5_H_7_N_2_)^+^ cations (Bélombé *et al.*, 2009; Nenwa *et al.*, 2010; Chérif *et al.*, 2011). The bond distances for the oxalate ions compare well with those reported for other oxalate complexes (Marinescu *et al.*, 2000) as do the main geometric parameters of the (C_6_H_9_N_2_)^+^ cation (Fun *et al.*, 2008, 2009, 2010; Jebas *et al.*, 2009; Quah *et al.*, 2008; Ramesh *et al.*, 2010; Rotondo *et al.*, 2009; Pan *et al.*, 2008; Chérif *et al.*, 2011). The cations are located between the anions (Fig. 2) and within the cation layer, the methyl groups of each pair of closest cations point in opposite directions. The crystal packing is stabilized by N—H···O and O—H···O hydrogen bonds (Blessing, 1986; Brown, 1976) between (C_6_H_9_N_2_)^+^ and [Cr(C_2_O_4_)_2_(H_2_O)_2_]^-^ involving the uncoordinated water molecule (O11) as both acceptor and donor and the coordinated water molecules (O1 and O2) only as donors. These interactions link the layers together forming a three-dimensional network and reinforcing the cohesion of the ionic structure.

### 2. Experimental

The title compound was prepared as good quality pink single crystals from a mixture of chromium (III) nitrate nonahydrate, 2-amino-6-methylpyridinium and oxalic acid in a 1:1:1 molar ratio in ethanol and water (50/50 v:v). The resulting mixture was heated to boiling and was stirred for one hour. After two weeks single pink crystals were obtained by slow evaporation at room temperature.

### 3. Refinement

The hydrogen atoms were located in difference Fourier maps. Those attached to carbon were placed in calculated positions (C—H = 0.93 - 0.96 Å) while those attached to nitrogen and oxygen were placed in the experimental positions and their coordinates adjusted to give N—H = 0.93 Å and O—H = 0.83 Å. All were included as riding contributions with isotropic displacement parameters 1.2 - 1.5 times those of the attached atoms.

### Crystal data

**Table:**

(C_6_H_9_N_2_)[Cr(C_2_O_4_)_2_(H_2_O)_2_]·H_2_O	*F*(000) = 1608
*M**_r_* = 391.24	*D*_x_ = 1.705 Mg m^−^^3^
Monoclinic, *C*2/*c*	Mo *K*α radiation, λ = 0.71073 Å
Hall symbol: -C 2yc	Cell parameters from 25 reflections
*a* = 18.572 (3) Å	θ = 10–15°
*b* = 11.025 (2) Å	µ = 0.81 mm^−^^1^
*c* = 14.975 (3) Å	*T* = 298 K
β = 96.28 (2)°	Prism, pink
*V* = 3047.8 (10) Å^3^	0.56 × 0.42 × 0.33 mm
*Z* = 8	

### Data collection

**Table:**

Enraf–Nonius CAD-4 diffractometer	2896 reflections with *I* > 2σ(*I*)
Radiation source: fine-focus sealed tube	*R*_int_ = 0.037
Graphite monochromator	θ_max_ = 27.0°, θ_min_ = 2.2°
non–profiled ω/2θ scans	*h* = −23→23
Absorption correction: ψ scan (North *et al.*, 1968)	*k* = −1→14
*T*_min_ = 0.679, *T*_max_ = 0.764	*l* = −19→3
4604 measured reflections	2 standard reflections every 120 min
3312 independent reflections	intensity decay: 3.2%

### Refinement

**Table:**

Refinement on *F*^2^	Primary atom site location: structure-invariant direct methods
Least-squares matrix: full	Secondary atom site location: difference Fourier map
*R*[*F*^2^ > 2σ(*F*^2^)] = 0.040	Hydrogen site location: mixed
*wR*(*F*^2^) = 0.112	H-atom parameters constrained
*S* = 1.07	*w* = 1/[σ^2^(*F*_o_^2^) + (0.0677*P*)^2^ + 2.2239*P*] where *P* = (*F*_o_^2^ + 2*F*_c_^2^)/3
3312 reflections	(Δ/σ)_max_ = 0.001
218 parameters	Δρ_max_ = 0.65 e Å^−^^3^
0 restraints	Δρ_min_ = −0.45 e Å^−^^3^

### Special details

**Table:**

Geometry. All esds (except the esd in the dihedral angle between two l.s. planes) are estimated using the full covariance matrix. The cell esds are taken into account individually in the estimation of esds in distances, angles and torsion angles; correlations between esds in cell parameters are only used when they are defined by crystal symmetry. An approximate (isotropic) treatment of cell esds is used for estimating esds involving l.s. planes.
Refinement. Refinement of *F*^2^ against ALL reflections. The weighted *R*-factor *wR* and goodness of fit *S* are based on *F*^2^, conventional *R*-factors *R* are based on *F*, with *F* set to zero for negative *F*^2^. The threshold expression of *F*^2^ > σ(*F*^2^) is used only for calculating *R*-factors(gt) *etc*. and is not relevant to the choice of reflections for refinement. *R*-factors based on *F*^2^ are statistically about twice as large as those based on *F*, and *R*- factors based on ALL data will be even larger.

### Fractional atomic coordinates and isotropic or equivalent isotropic displacement parameters (Å^2^)

**Table:**

	*x*	*y*	*z*	*U*_iso_*/*U*_eq_	
Cr1	0.32255 (2)	0.27746 (3)	0.19839 (2)	0.02219 (13)	
O1	0.38682 (8)	0.29143 (13)	0.09943 (9)	0.0291 (3)	
H3	0.3720	0.2466	0.0568	0.044*	
H4	0.4279	0.2639	0.1150	0.044*	
O2	0.25655 (9)	0.26707 (13)	0.29364 (10)	0.0325 (3)	
H2	0.2587	0.3260	0.3284	0.049*	
H10	0.2578	0.2050	0.3251	0.049*	
O3	0.24053 (8)	0.26015 (12)	0.10486 (9)	0.0259 (3)	
O4	0.40928 (8)	0.28451 (13)	0.28656 (10)	0.0315 (3)	
O5	0.29813 (8)	0.45160 (12)	0.18501 (10)	0.0290 (3)	
O6	0.33921 (8)	0.10332 (13)	0.20991 (9)	0.0284 (3)	
O7	0.15703 (8)	0.37130 (15)	0.02425 (10)	0.0379 (4)	
O8	0.22682 (10)	0.57497 (14)	0.09691 (10)	0.0374 (4)	
O9	0.40914 (10)	−0.03256 (15)	0.28778 (13)	0.0468 (4)	
O10	0.49100 (8)	0.16363 (17)	0.36144 (11)	0.0389 (4)	
O11	0.24742 (10)	−0.05094 (17)	0.09548 (11)	0.0480 (5)	
H1	0.2610	−0.0620	0.0451	0.072*	
H11	0.2725	0.0015	0.1239	0.072*	
N1	0.49218 (10)	0.78135 (16)	0.37925 (13)	0.0325 (4)	
H7	0.4632	0.8434	0.3532	0.039*	
N2	0.56211 (12)	0.93977 (19)	0.43928 (15)	0.0455 (5)	
H5	0.6002	0.9646	0.4807	0.055*	
H13	0.5320	0.9978	0.4096	0.055*	
C1	0.21055 (11)	0.35961 (17)	0.07919 (12)	0.0246 (4)	
C2	0.43552 (11)	0.1812 (2)	0.31024 (13)	0.0281 (4)	
C3	0.24748 (11)	0.47407 (17)	0.12350 (13)	0.0254 (4)	
C4	0.39248 (11)	0.07128 (19)	0.26753 (14)	0.0288 (4)	
C5	0.52196 (14)	0.5768 (2)	0.4038 (2)	0.0480 (6)	
H12	0.5124	0.4947	0.3949	0.058*	
C6	0.58452 (15)	0.6150 (3)	0.4566 (2)	0.0518 (7)	
H9	0.6165	0.5570	0.4830	0.062*	
C7	0.59978 (14)	0.7335 (3)	0.47029 (18)	0.0449 (6)	
H6	0.6416	0.7570	0.5061	0.054*	
C8	0.47476 (13)	0.6629 (2)	0.36524 (17)	0.0397 (5)	
C9	0.40503 (15)	0.6364 (3)	0.3091 (2)	0.0587 (8)	
H8	0.3659	0.6395	0.3460	0.088*	
H14	0.4072	0.5570	0.2832	0.088*	
H15	0.3971	0.6956	0.2621	0.088*	
C10	0.55186 (12)	0.8210 (2)	0.42989 (15)	0.0337 (5)	

### Atomic displacement parameters (Å^2^)

**Table:**

	*U*^11^	*U*^22^	*U*^33^	*U*^12^	*U*^13^	*U*^23^
Cr1	0.02305 (19)	0.01884 (19)	0.02292 (19)	0.00059 (11)	−0.00543 (12)	0.00012 (10)
O1	0.0281 (7)	0.0301 (7)	0.0279 (7)	−0.0021 (6)	−0.0021 (6)	−0.0019 (6)
O2	0.0400 (8)	0.0271 (8)	0.0304 (8)	0.0037 (6)	0.0047 (6)	0.0027 (6)
O3	0.0260 (7)	0.0220 (7)	0.0278 (7)	−0.0003 (5)	−0.0059 (5)	−0.0012 (5)
O4	0.0305 (8)	0.0311 (8)	0.0302 (8)	−0.0023 (6)	−0.0088 (6)	−0.0015 (6)
O5	0.0329 (7)	0.0200 (6)	0.0317 (7)	0.0001 (6)	−0.0071 (6)	−0.0014 (5)
O6	0.0272 (7)	0.0228 (7)	0.0330 (7)	0.0015 (6)	−0.0062 (6)	0.0006 (6)
O7	0.0356 (8)	0.0361 (8)	0.0377 (8)	0.0107 (7)	−0.0152 (7)	−0.0085 (7)
O8	0.0545 (10)	0.0220 (7)	0.0333 (8)	0.0083 (7)	−0.0054 (7)	0.0007 (6)
O9	0.0468 (10)	0.0309 (9)	0.0602 (11)	0.0099 (7)	−0.0058 (8)	0.0134 (8)
O10	0.0261 (7)	0.0521 (10)	0.0359 (8)	0.0011 (7)	−0.0087 (6)	0.0098 (7)
O11	0.0649 (12)	0.0413 (10)	0.0375 (9)	−0.0201 (9)	0.0042 (8)	0.0002 (7)
N1	0.0279 (9)	0.0289 (9)	0.0396 (10)	0.0063 (7)	−0.0012 (7)	0.0018 (7)
N2	0.0462 (12)	0.0356 (10)	0.0534 (12)	−0.0063 (9)	−0.0005 (9)	−0.0046 (9)
C1	0.0270 (9)	0.0249 (10)	0.0214 (9)	0.0032 (7)	−0.0002 (7)	−0.0016 (7)
C2	0.0229 (9)	0.0371 (11)	0.0240 (9)	0.0005 (8)	0.0003 (7)	0.0042 (8)
C3	0.0316 (10)	0.0229 (9)	0.0215 (9)	0.0017 (7)	0.0015 (7)	−0.0015 (7)
C4	0.0264 (10)	0.0280 (10)	0.0312 (10)	0.0029 (8)	0.0005 (8)	0.0053 (8)
C5	0.0487 (15)	0.0287 (11)	0.0673 (17)	0.0031 (11)	0.0094 (13)	0.0012 (11)
C6	0.0435 (14)	0.0473 (14)	0.0639 (17)	0.0152 (12)	0.0029 (12)	0.0120 (13)
C7	0.0331 (12)	0.0506 (15)	0.0486 (14)	0.0068 (10)	−0.0061 (11)	0.0036 (11)
C8	0.0338 (11)	0.0353 (12)	0.0509 (14)	−0.0016 (9)	0.0087 (10)	−0.0066 (10)
C9	0.0378 (13)	0.0634 (19)	0.0733 (19)	−0.0083 (13)	−0.0011 (13)	−0.0253 (15)
C10	0.0295 (10)	0.0381 (12)	0.0334 (11)	0.0027 (9)	0.0028 (8)	0.0033 (9)

### Geometric parameters (Å, º)

**Table:**

Cr1—O6	1.9493 (15)	N1—C10	1.346 (3)
Cr1—O3	1.9626 (14)	N1—C8	1.357 (3)
Cr1—O4	1.9695 (15)	N1—H7	0.9300
Cr1—O5	1.9778 (15)	N2—C10	1.328 (3)
Cr1—O2	1.9830 (15)	N2—H5	0.9300
Cr1—O1	2.0080 (15)	N2—H13	0.9300
O1—H3	0.8300	C1—C3	1.550 (3)
O1—H4	0.8300	C2—C4	1.551 (3)
O2—H2	0.8299	C5—C8	1.375 (4)
O2—H10	0.8300	C5—C6	1.397 (4)
O3—C1	1.270 (2)	C5—H12	0.9300
O4—C2	1.274 (3)	C6—C7	1.348 (4)
O5—C3	1.267 (2)	C6—H9	0.9300
O6—C4	1.289 (2)	C7—C10	1.404 (3)
O7—C1	1.226 (2)	C7—H6	0.9300
O8—C3	1.229 (2)	C8—C9	1.494 (4)
O9—C4	1.216 (3)	C9—H8	0.9600
O10—C2	1.231 (2)	C9—H14	0.9600
O11—H1	0.8299	C9—H15	0.9600
O11—H11	0.8301		
			
O6—Cr1—O3	94.05 (6)	O7—C1—C3	119.34 (17)
O6—Cr1—O4	82.49 (6)	O3—C1—C3	114.48 (16)
O3—Cr1—O4	175.08 (6)	O10—C2—O4	125.7 (2)
O6—Cr1—O5	175.89 (6)	O10—C2—C4	119.50 (19)
O3—Cr1—O5	82.57 (6)	O4—C2—C4	114.82 (16)
O4—Cr1—O5	101.03 (6)	O8—C3—O5	126.40 (19)
O6—Cr1—O2	89.09 (6)	O8—C3—C1	119.36 (18)
O3—Cr1—O2	90.87 (7)	O5—C3—C1	114.24 (16)
O4—Cr1—O2	92.56 (7)	O9—C4—O6	125.5 (2)
O5—Cr1—O2	88.63 (6)	O9—C4—C2	121.83 (19)
O6—Cr1—O1	92.26 (6)	O6—C4—C2	112.65 (17)
O3—Cr1—O1	87.62 (6)	C8—C5—C6	118.9 (2)
O4—Cr1—O1	89.03 (6)	C8—C5—H12	120.6
O5—Cr1—O1	89.94 (6)	C6—C5—H12	120.6
O2—Cr1—O1	178.04 (6)	C7—C6—C5	121.7 (2)
Cr1—O1—H3	110.3	C7—C6—H9	119.1
Cr1—O1—H4	111.5	C5—C6—H9	119.1
H3—O1—H4	102.5	C6—C7—C10	119.2 (2)
Cr1—O2—H2	114.5	C6—C7—H6	120.4
Cr1—O2—H10	118.3	C10—C7—H6	120.4
H2—O2—H10	107.0	N1—C8—C5	118.0 (2)
C1—O3—Cr1	114.35 (12)	N1—C8—C9	116.9 (2)
C2—O4—Cr1	114.29 (13)	C5—C8—C9	125.1 (2)
C3—O5—Cr1	114.03 (12)	C8—C9—H8	109.5
C4—O6—Cr1	115.59 (13)	C8—C9—H14	109.5
H1—O11—H11	111.1	H8—C9—H14	109.5
C10—N1—C8	124.6 (2)	C8—C9—H15	109.5
C10—N1—H7	113.7	H8—C9—H15	109.5
C8—N1—H7	121.7	H14—C9—H15	109.5
C10—N2—H5	116.8	N2—C10—N1	118.7 (2)
C10—N2—H13	123.8	N2—C10—C7	123.7 (2)
H5—N2—H13	119.3	N1—C10—C7	117.6 (2)
O7—C1—O3	126.18 (18)		
			
Cr1—O3—C1—O7	−177.14 (17)	O4—C2—C4—O9	−176.0 (2)
Cr1—O3—C1—C3	3.0 (2)	O10—C2—C4—O6	−174.65 (18)
Cr1—O4—C2—O10	176.81 (17)	O4—C2—C4—O6	4.3 (3)
Cr1—O4—C2—C4	−2.0 (2)	C8—C5—C6—C7	−0.3 (4)
Cr1—O5—C3—O8	−173.21 (18)	C5—C6—C7—C10	−0.5 (4)
Cr1—O5—C3—C1	6.1 (2)	C10—N1—C8—C5	−1.4 (4)
O7—C1—C3—O8	−6.7 (3)	C10—N1—C8—C9	178.2 (2)
O3—C1—C3—O8	173.12 (19)	C6—C5—C8—N1	1.2 (4)
O7—C1—C3—O5	173.94 (18)	C6—C5—C8—C9	−178.4 (3)
O3—C1—C3—O5	−6.2 (2)	C8—N1—C10—N2	−179.0 (2)
Cr1—O6—C4—O9	175.95 (19)	C8—N1—C10—C7	0.7 (4)
Cr1—O6—C4—C2	−4.4 (2)	C6—C7—C10—N2	−180.0 (2)
O10—C2—C4—O9	5.1 (3)	C6—C7—C10—N1	0.3 (4)

### Hydrogen-bond geometry (Å, º)

**Table:**

*D*—H···*A*	*D*—H	H···*A*	*D*···*A*	*D*—H···*A*
O1—H3···O7^i^	0.83	1.82	2.643 (2)	171
O1—H4···O10^ii^	0.83	1.87	2.681 (2)	165
O2—H2···O11^iii^	0.83	1.78	2.611 (2)	173
O2—H10···O8^iv^	0.83	1.85	2.675 (2)	172
N1—H7···O9^v^	0.93	1.90	2.829 (2)	174
N2—H5···O7^vi^	0.93	2.16	2.928 (3)	139
N2—H13···O10^v^	0.93	2.08	2.976 (3)	162
C9—H8···O3^iii^	0.96	2.56	3.405 (3)	147
C9—H14···O5	0.96	2.64	3.277 (3)	124
O11—H1···O8^i^	0.83	2.17	2.983 (2)	167
O11—H11···O6	0.83	2.03	2.845 (2)	169

Symmetry codes: (i) −*x*+1/2, −*y*+1/2, −*z*; (ii) −*x*+1, *y*, −*z*+1/2; (iii) −*x*+1/2, *y*+1/2, −*z*+1/2; (iv) −*x*+1/2, *y*−1/2, −*z*+1/2; (v) *x*, *y*+1, *z*; (vi) *x*+1/2, −*y*+3/2, *z*+1/2.
